# A meta-analysis: dietary carbohydrates do not increase body fat or fasted insulin and glucose in cats

**DOI:** 10.1093/jas/skaf071

**Published:** 2025-03-07

**Authors:** Hannah Godfrey, Jennifer L Ellis, Adronie Verbrugghe

**Affiliations:** Department of Biomedical Sciences, Ontario Veterinary College, University of Guelph, Guelph, ON, Canada N1G 2W1; Department of Animal Biosciences, Ontario Agricultural College, University of Guelph, Guelph, ON, Canada N1G 2W1; Department of Clinical Studies, Ontario Veterinary College, University of Guelph, Guelph, ON, Canada N1G 2W1; Department of Animal Biosciences, Ontario Agricultural College, University of Guelph, Guelph, ON, Canada N1G 2W1

**Keywords:** Feline, nitrogen-free extract, macronutrients, body composition, glucose tolerance, insulin sensitivity

## Abstract

Commercial cat foods contain a greater carbohydrate content, such as nitrogen-free extract (**NFE**), compared to a typical prey species. This has led to postulations that increased carbohydrate intake is causing a rise in obesity and IR in cats. Studies investigating high-carbohydrate diets on insulin and glucose responses show inconsistent results. A meta-analysis using 16 studies was conducted to elucidate the relationship between NFE content and body fat mass (**BFM**, *n* = 9), fasted insulin (*n* = 12), and fasted glucose concentrations (*n* = 14). Dietary NFE, fat, and protein content (% metabolizable energy), as well as daily energy intake (**DEI**), body weight, body condition (lean, obese), and study design metrics were considered as fixed effects in univariate and multivariate models using proc mixed in SAS, treating study as a random effect. Model evaluation was conducted using corrected Akaike Information Criteria, concordance correlation coefficient, and the root mean square prediction error. The best-fitting model for BFM was the interaction between NFE content and DEI, predicting BFM to decrease when NFE content increased as a proportion of the DEI (*P* < 0.05). From univariate models, fasted insulin was positively associated with BFM and dietary fat content (*P* < 0.05), whereas an increase in NFE content was associated with a decrease in fasted insulin in a subgroup of studies (*n* = 6) of only lean cats (*P* < 0.05). No significance was observed for models predicting fasted glucose from diet or body composition variables (*P* > 0.05). The results of this meta-analysis indicate that dietary carbohydrates (NFE), included between 2.8% and 57% ME, are not a risk factor for greater BFM, fasted insulin, and glucose concentrations in cats, suggesting that NFE does not pose a risk for feline obesity, IR, or hyperglycemia. However, future studies should consider postprandial responses of insulin and glucose to macronutrient compositions to further investigate the role of dietary carbohydrates on IR in cats, with particular attention to the role of dietary fat, and the role of body condition.

## Teaser

A meta-analysis was conducted to investigate the role of dietary carbohydrates on body fat mass, fasted insulin, and fasted glucose in cats. The findings seem to contradict current hypotheses that suggest that increased dietary carbohydrates are a risk factor for obesity and insulin resistance in cats.

## Graphical Abstract

**Graphical Abstract:**
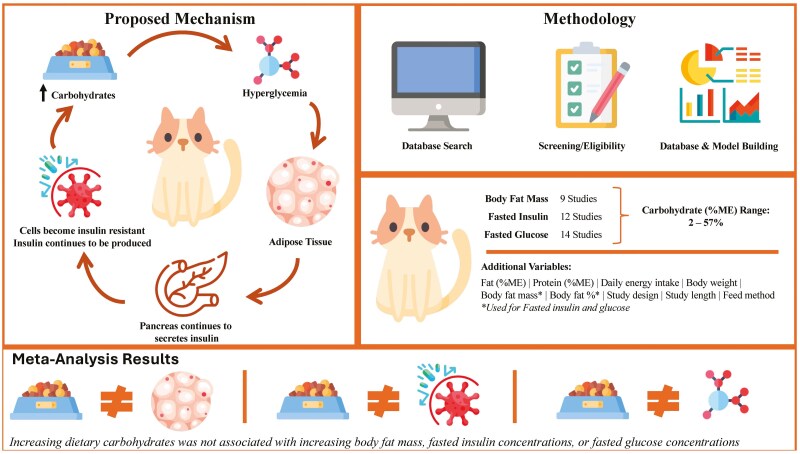

## 1. Introduction

Since domestication, the diet for cats, an obligate carnivore, has shifted from that of the natural prey species to commercial extruded dry foods. As an obligate carnivore, cats cannot meet all of their specific nutrient requirements through the consumption of only plant material. Rather, they require specific nutrients through consumption of animal tissues. A typical prey species for a feral cat consists of 52% metabolizable energy (**ME**) crude protein, 46% ME crude fat, and 2% ME carbohydrates, represented as nitrogen-free extract (**NFE**) (Plantinga et al., 2011). The low carbohydrate intake from the natural diet aligns with the nutritional idiosyncrasies in the cat such as a lack of, or reduced, digestive enzyme capacity, limited length of the intestine, and lack of glucokinase activity in the liver (Kienzle, 1993a, 1993b; Tanaka et al., 2005; Hiskett et al., 2009; Shirazi-Beechey et al., 2011; Verbrugghe and Hesta, 2017). Commercial cat foods can contain up to 156 g/1,000 kcal NFE without risking nutrient imbalances for protein or fat according to their minimum Association of American Feed Control Officials (**AAFCO**) recommendations for adult maintenance (AAFCO, 2024). In the UK, it was reported that extruded dry commercial cat foods contained an average of 36% of ME from crude protein, 28% ME from crude fat, and 36% ME from NFE on a dry matter basis (Davies et al., 2017). The addition of carbohydrates (NFE) to cat foods contributes to structural properties and aids in the processing and preservation of the kibble and wet foods (Case et al., 2010; Villaverde and Fascetti, 2014). Additionally, carbohydrates can be a source of energy in cat diets despite the proposed hypotheses that cats have a reduced capacity to digest, absorb, and metabolize carbohydrates (Morris et al., 1977; Kienzle, 1993c; de-Oliveira et al., 2008; Verbrugghe and Hesta, 2017). Regardless, the metabolic differences in cats, an obligate carnivore, have led to postulations that commercial diets, which contain moderate or high dietary carbohydrate inclusions compared to that of the typical prey species, negatively affect feline health, specifically, to cause obesity, insulin resistance (**IR**), or diabetes mellitus (Buffington, 2008; Villaverde and Fascetti, 2014; Rowe et al., 2015).

Simply put, obesity is characterized as excessive adipose tissue resulting from a positive energy balance. Multiple risk factors contribute to obesity in domestic cats including, but not limited to, ad libitum feeding regimens, sex and neuter status, indoor confinement, and of particular interest, feeding a primarily dry food diet (Scarlett et al., 1994; Lund et al., 2005; Buffington, 2008; Colliard et al., 2009; Rowe et al., 2015). IR in cats can be characterized as a reduced sensitivity to insulin ultimately resulting in a decreased rate of disposal of glucose (Curry et al., 1982; Feldhahn et al., 1999; Mori et al., 2008; Verbrugghe et al., 2012). Overall, this can be attributed to the inability of insulin to bind to insulin receptors or due to disruptions in the signal transduction pathway when insulin is bound and is often a consequence of obesity (Curry et al., 1982; Feldhahn et al., 1999; Mori et al., 2008; Verbrugghe et al., 2012). Indeed, obese cats have less expression of insulin-sensitive transporters and insulin signaling genes (Mori et al., 2008). Previously, cats fed a high-carbohydrate diet (NFE = 47% ME) exhibited prolonged hyperglycemia following a meal-test, suggestive of alterations in insulin sensitivity (Farrow et al., 2013). However, neither IR nor a reduction in the effectiveness of glucose was observed in these cats in that study when evaluated using an intravenous glucose tolerance test. Alternatively, high-fat diets increase energy intake and body weight (**BW**) gain attributed to increases in body fat mass (**BFM**) compared to high-carbohydrate diets when fed ad libitum and could be a greater risk factor for obesity in cats (Nguyen et al., 2004; Backus et al., 2007; Martin et al., 2010). Additional considerations wherein amino acids, rather than glucose, are thought to be more potent stimulators of insulin secretion in cats should also be made (Curry et al., 1982).

Conflicting results have been reported in the literature. Farrow et al., observed increases in glucose and insulin with a high-carbohydrate diet compared to a high-fat or high-protein diets (Farrow et al., 2013); though Leray et al., observed no differences between high-carbohydrate and high-protein diets which were repeated by Hoenig et al. (Leray et al., 2006; Hoenig et al., 2007a). Overall, the role of dietary carbohydrates in commercial cat food for obesity and IR in cats is not well understood leading to questions regarding whether increasing dietary carbohydrates, as NFE, in cat foods can increase BFM and pose a risk for obesity onset, as well as whether dietary carbohydrates are strong influencers of blood glucose and insulin. It is hypothesized that increasing dietary carbohydrates does not affect BFM in cats. Rather, feeding at an amount greater than the cats’ energy requirements, often driven by dietary fats, leads to BFM gain. Previously, higher basal insulin concentrations in cats were associated with a greater likelihood of developing glucose intolerance, and an elevated fasted glucose concentration indicated dysfunction of the β cells (Ferrannini, 1997; Appleton et al., 2001). Thus, fasted insulin and glucose concentrations could indicate changes in insulin sensitivity in cats. Due to the unique metabolism in cats wherein amino acids are considered more potent stimulators of insulin secretion, it is hypothesized that changes in dietary NFE content are not a good predictor of fasted insulin or glucose concentrations. A meta-analysis approach allows for synthesis of findings across multiple studies to answer research questions with increased statistical power and could be beneficial for answering the present research question. Therefore, the objectives of this study were to use a meta-analysis approach to determine the relationship between dietary carbohydrates and BFM in cats, and to investigate the effect of dietary carbohydrates on fasted glucose and insulin concentrations in cats. For the purposes of this meta-analysis, carbohydrates will refer to the NFE.

## 2. Materials and Methods

### 2.1. Literature search and database development

**Figure 1.**
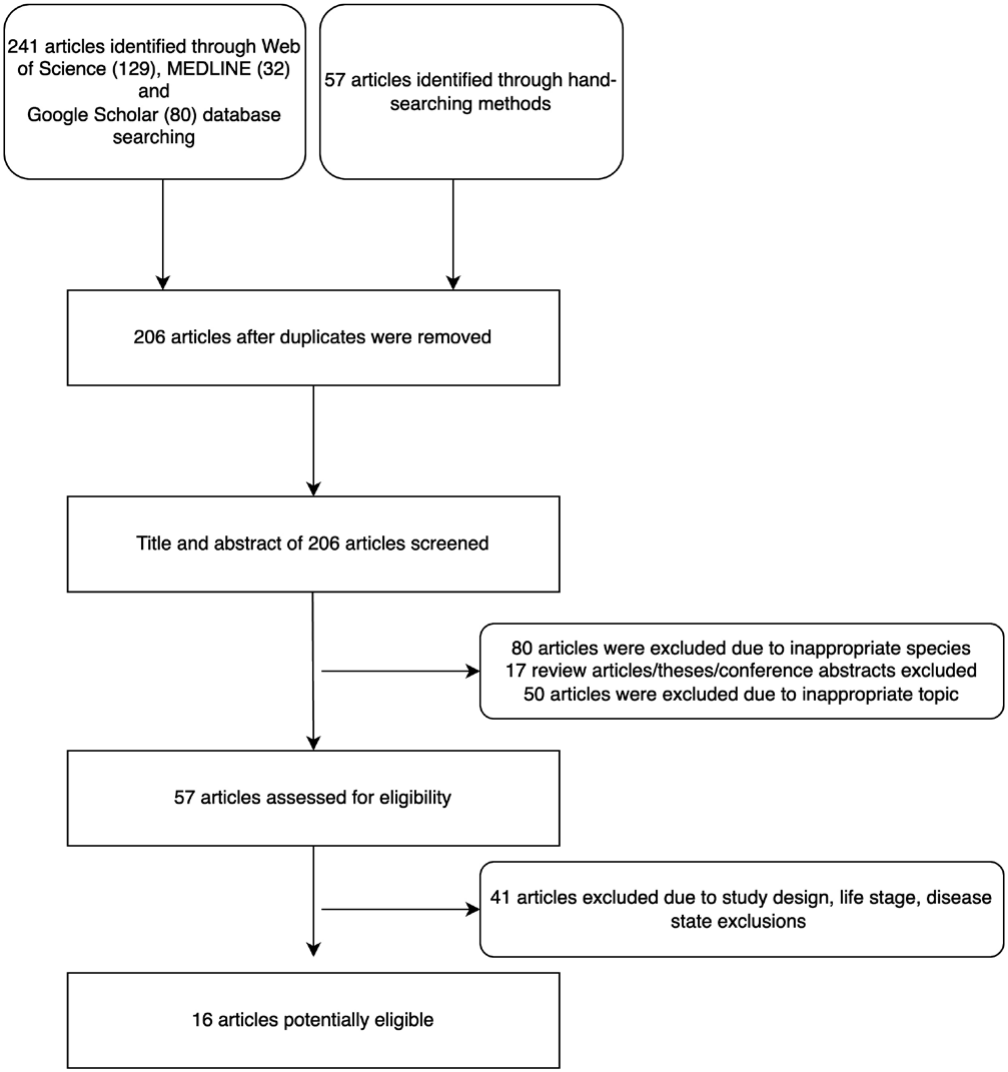
Literature funnel of systematic review to assess dietary carbohydrate content on BFM, fasted insulin, and fasted glucose.

In January 2024, a systematic literature search was conducted via Google Scholar, Medline, and the Web of Science databases. No restrictions were placed for publication years. Keyword combinations following Boolean Logic search string were employed to search for papers as follows: (cats OR cat OR feline OR “felis catus” NOT rat NOT mice NOT dog) AND (carbohydrate OR “nitrogen free extract” OR “nitrogen-free extract” OR NFE OR macronutrients) AND (“weight gain” OR “body weight” OR “total tissue mass” OR “body mass” OR “body composition”) AND (“fat mass” OR “fat gain” OR “body composition”) AND (insulin OR “serum insulin” OR “plasma insulin” OR “blood glucose” OR “serum glucose” OR “blood glucose” OR “glucose tolerance” OR “insulin resistance” OR “insulin sensitivity” OR “glucose curve” OR “glucose intolerance” OR disglycemia OR dysglycemia OR glycemia OR disglycaemia OR dysglycaemia OR glycemia). Hand-searching of multiple published review papers returned from using the Boolean Logic string terms on the relevant topics (Verbrugghe et al., 2012; Villaverde & Fascetti, 2014; Sparkes et al., 2015; Clark and Hoenig, 2021; Laflamme et al., 2022) was also utilized. A total of 129 records were retrieved from Web of Science, 32 from MEDLINE, and 80 from Google Scholar with an additional 57 records retrieved through hand-searching (Fig. 1). Duplicates were then removed to form the initial dataset (*n* = 206). Title and abstracts were screened for eligibility resulting in 57 articles which were then assessed for exclusion/inclusion criteria.

Upon screening, papers were excluded from analysis if they were not primary research articles (i.e., conference abstract, review article, book chapter, academic theses). “Cat” or “feline” was required to be included in the title, abstract, or keywords. Additional exclusions included any health conditions, except for obese condition, such as cats with a diabetes mellitus diagnosis. For studies to be considered eligible, a minimum of 2 test diets had to be investigated over a minimum of 2 wk, and a minimum of 2 diets differing in NFE content had to have been tested such that one diet partially replaced protein or fat, or a dilution of both protein and fat, with NFE as a percent of the ME. Growth (<6 mo of age), pregnancy, and lactation were considered exclusions. This resulted in 16 studies in the total dataset where 9 studies measured BFM, 12 measured fasted insulin concentrations, and 14 measured fasted glucose concentrations, and these are summarized in Table 1.

**Table 1.** Summary of publications used to assess dietary carbohydrate (NFE) content impact on BFM, fasted insulin, and fasted glucose

Reference	Study design^1^	*N* ^2^	Study length^3^	Feeding method^4^	BC^5^	Sex	Neuter status^6^	Age (yr)^7^	Trade^8^	Number of test diets	BFM^9^	Fasted insulin	Fasted glucose
Farrow et al. (2013)	P	24	5	Ad lib	Lean	Mix	Gx	2 to 6	Dilution	3	—	X	X
Tan et al. (2011)	P	31	4	MER	Lean	Mix	Gx	2 to 4	NFE-Protein	2	X	X	X
Gooding et al. (2015)	P	20	12	MER	Lean	Mix	n/a	3.5 ± 0.5	NFE-Fat	2	X	X	X
Coradini et al. (2011)	P	31	4	MER	Lean	Mix	Gx	2 to 4	NFE-Protein	2	X	X	X
	P	31	8	Ad Lib	Lean				NFE-Protein	2	X	X	X
Wei et al. (2011)	P	11	17	Ad Lib	Obese	Mix	Gx	4.3 ± 1.2	NFE-Protein	2	X	X	X
Martin et al. (2010)	C	10	6	MER	Mixed	Mix	Gx	7 to 8	Dilution	3	—	X	X
Backus et al. (2007)	P	48	17	Ad Lib	Lean	Mix	Gx	9 to 12^10^	NFE-fat	4	X	X	X
Hoenig et al. (2007a)	C	12	17	MER	Lean	Mix	Gx	4.3 ± 0.4	NFE-Protein	2	X	—	—
	C	16	17	MER	Obese			5.1 ± 1.2	NFE-Protein	2	X	—	—
Verbrugghe et al. (2010)	C	9	3	MER	Lean	Mix	Mix	3 to 10	Dilution	3	—	X	X
Des Courtis et al. (2015)	P	16	8	Restrict	Obese	Mix	Mix	5.2 ± 1.65	NFE-Protein	2	X	X	X
Li and Pan (2020)	P	19	8	MER	Lean	Mix	Gx	7.2^11^	NFE-Protein	2	X	—	—
	P	20	8	MER	Obese				NFE-Protein	2	X	—	—
Gooding et al. (2014)	C	10	2	MER	Lean	Mix	Gx	10 ± 2^10^	NFE-Fat	2	—	X	X
Thiess et al. (2004)	C	6	3	MER	Lean	Male	Mix	19 to 21^10^	NFE-Fat	2	—	X	X
Laflamme and Hannah (2005)	P	16	26	Restrict	Obese	Female	Mix	N/A	NFE-Protein	2	X	—	X
Deng et al. (2013)	C	12	2	MER	Lean	Male	Gx	3	NFE-Fat	2	—	X	X
	C	12	2	MER	Lean				Dilution	2	—	X	X
Musco et al. (2017)	C	6	4	MER	Lean	Mix	Gx	3.5 ± 0.2	Dilution	4	—	—	X

^1^P = parallel, C = cross-over.

^2^ *N* = sample size (number of cats).

^3^Given in weeks.

^4^Ad lib = ad libitum, MER = maintenance energy requirement.

^5^Body composition.

^6^Gx = gonadectomized, n/a = not available, Mix = intact and gonadectomized.

^7^Age is provided in range or as mean ± SEM.

^8^NFE-protein = nitrogen-free extract replaced by protein, NFE-fat = nitrogen-free extract replaced by fat, Dilution = nitrogen-free extract replaced by both fat and protein.

^9^BFM = body fat mass, X = Measured.

^10^Given in months.

^11^Given as median.

Extracted data from each study included sample size, study design (i.e., parallel or cross-over design), sex (male or female), body composition (**BC**) (lean or obese assessed by body condition score or other validated analysis), mean BW, as well as neuter status. Study length and feeding management (i.e., ad libitum feeding, calorie restriction, or feeding to maintenance energy requirements) were included. The variable BFM was added when applicable and body fat percentage (BF%) was calculated as the mean proportion of fat mass divided by the mean BW as a percentage. Dietary macronutrients of each test diet within each study were included on a % ME basis. For the purposes of the analysis, carbohydrate was represented as the NFE calculated using the Modified Atwater Equation (National Research Council, 2006):


$$\begin{array}{lll} NFE=\; & 100-(crude\;protein+crude\;fat\; & +crude\;f\;\;iber+ash+moisture) \end{array}$$

For the dataset, all 3 macronutrients were inputted as % ME using the Modified Atwater factors; 3.5, 3.5, 8.5 for protein, NFE, and fat, respectively (National Research Council, 2006). Daily energy intake (**DEI**) was converted to kcal for all studies and presented as kcal/kg BW per day where the mean BW for the respective group was used to calculate the energy on a kg BW basis. Fasted glucose (mmol/L) and fasted insulin concentrations (pmol/L) were included in the dataset and included serum, plasma, and whole blood measures. In cases where data was provided via graph, Web Plot Digitizer (WebPlotDigitizer, version 4.7) was used to extract the values.

Further categorization was made to account for NFE, fat, and protein contents of the diets tested. First, the NFE, fat, and protein content of each test diet was assessed and allocated as high (NFE: > 30% ME; Fat: > 35% ME; Protein: > 42% ME), moderate (NFE: 24% ME—30% ME; Fat: 28% ME—35% ME; Protein: 34% ME—42% ME), or low (NFE: < 24% ME; Fat: < 28% ME; Protein: < 34% ME) to create a “Level” variable for NFE, fat, and protein, respectively. A variable “Trade” was made to identify when studies replaced protein for NFE (% ME) and when fat was used to replace NFE (% ME). An additional “Trade” category was made in cases where fat and protein were both altered for an increase or decrease in NFE content (% ME). This variable was used to factor whether replacement of fat or protein for NFE (NFE-fat and NFE-protein, respectively), or both (Dilution) influenced the outcome variable. Summary statistics (mean, median, minimum, and maximum) for the continuous variables and 3 response variables, BFM, fasted glucose concentrations, and fasted insulin concentrations, from the final dataset are summarized in Table 2.

**Table 2.** Summary statistics for continuous driving (X) variables and response variables used in the final dataset of the meta-analysis

Summary statistics	NFE^1^, % ME	Fat, % ME	Protein, % ME	DEI, kcal/kg BW	BW, kg	BFM, kg	BF%, %	Study Length, wk	Glucose, mmol/L	Insulin, pmol/L
*N* ^2^	53	53	53	49	53	29	*29*	53	45	39
Mean	29.0	35.2	35.1	59.0	4.6	1.3	26.7	9.0	4.6	54.5
SD	14.2	11.9	9.1	24.8	0.9	0.9	12.1	6.7	0.6	19.6
Median	27.0	34.9	33.0	51.4	4.7	1.0	25.6	6.0	4.8	40.2
Minimum	2.8	9.1	21.0	31.4	3.2	0.4	8.3	2.0	3.4	17.0
Maximum	57.0	63.5	54.2	151.7	6.3	3.6	58.7	26.0	5.9	162.0

^1^NFE = nitrogen-free extract; BF%=body fat percent; BFM = body fat mass; BW = body weight; DEI = daily energy intake; ME = metabolizable energy.

^2^ *N* = sample size, SD = standard deviation.

### 2.2. Model development

The following procedures were performed using SAS Studio (SAS Institute, Cary, NC). Driving variables (X) assessed for modeling BFM (Y variable) included the following continuous variables: NFE (% ME), fat (% ME), protein (% ME), DEI (kcal/kg BW), and the following categorical variables: Trade (NFE-fat; NFE-protein), study design (parallel or cross-over), and feeding method (ad libitum or to maintenance requirements). For the categorical variable, Trade, data were only available for NFE-fat and NFE-protein and no data were available for the Dilution. Regarding modeling of fasted glucose (mmol/L) and fasted insulin (pmol/L) the following continuous X variables were assessed: NFE (% ME), fat (% ME), protein (% ME), DEI (kcal/kg BW), BW (kg), BFM (kg), BF% (%), and study length (weeks). Additionally, the following categorical variables were considered: Trade (NFE for fat; NFE for protein; dilution of fat and protein for NFE), NFE Level (high, moderate, low), fat level (high, moderate, low), Protein Level (high, moderate, low), BC (lean; obese), study design (parallel or cross-over), and feeding method (ad libitum or to maintenance requirements). The PROC CORR procedure was used to evaluate the collinearity between the continuous X variables. For categorical X variables, a Kruskal–Wallis test was performed to observe collinearity between categorical and continuous X variables whereas the PROC FREQ and Chi-Square test was used to evaluate collinearity between categorical X variables. Continuous and categorical X variables that were considered collinear (*P < *0.05) were not considered for the same equation together, to avoid issues around multicollinearity.

The PROC MIXED procedure was used to assess mixed models within SAS Studio. To account for between study differences, study was considered a random effect (St-Pierre, 2001). Models were developed for each response variable: BFM, fasted insulin, and fasted glucose. First, univariate models were developed for each response variable using each individual X variable. Univariate models where NFE (% ME), Trade, or NFE Level was used were followed by the addition of X variables to build multivariate models for each response variable. Due to the limited number of studies assessing BFM or FM% and fasted insulin, and assessing BFM or FM% and fasted glucose, 2 approaches were used. One where BFM and FM% were considered and another where BFM and FM% were not considered in the multivariate models for fasted insulin and glucose.

Between and within model comparisons to indicate goodness of fit for identification of optimal variance/covariance structure and the inclusion or exclusion of fixed effects and random effects was completed by assessment of the corrected Akaike Information Criteria (AICc) during model development. A lower AICc value when comparing models was indicative of a better fit. A Cook’s distance test (> 4/*n*, where *n* represents the number of observations) within the PROC MIXED procedure was utilized for detection of outliers and removal of outliers was conducted in a sequential manner where applicable (Cook, 1977). Evaluation of model assumptions for residual and random effects distribution (normality) were conducted using visual assessment of QQ plots, histogram, and via the Shapiro-Wilk test. Significance of fixed effects was assessed at *P* < 0.05, and trends were discussed when the *P*-value was between 0.05 and 0.10.

For all equations for BFM, fasted insulin and fasted glucose, lean and obese cats were included; however, a subgroup analysis was also conducted determining the effects of NFE on BFM in lean cats only. This subgroup analysis was undertaken to minimize potential confounding effects associated with BFM variability and to provide clearer insights into whether NFE intake constitutes a risk factor for increasing BFM. Univariate models with NFE (% ME), protein (% ME), and fat (% ME) were developed. Due to the limited number of observations, multivariate models within the subgroup analysis were not developed.

### 2.3. Model evaluation

A visual and statistical assessment of the top 5 models for each response variable developed were conducted using SAS (SAS Institute, Cary, NC). First, plots using PROC SGPLOT were used to visually assess patterns, precision, and accuracy of the observed compared to the adjusted predicted values and patterns, bias, and homogeneity of the adjusted predicted compared to conditional residuals, calculated as predicted minus the observed value.

To statistical evaluate the developed model, multiple metrics were utilized. For an estimation of the overall prediction error, the mean square prediction error (MPSE) was calculated according to (Bibby and Toutenburg, 1977):


$$\begin{array}{l} \begin{array}{l} MSPE=\;\frac{\overset{n}{\underset{i=1}{\sum }}\;{\left(Y_{Obs}-\;Y_{Pred}\right)}^{2}}{n}\; \end{array} \end{array}$$ (1)

where *n* = total number of observations, *Y*_Obs_ = observed value, and *Y*_Prod_ = predicted value.

The MSPE was further expressed as the square root of MSPE (RMSPE) and relative to the observed mean to aid interpretation. Decomposition of the MSPE into error due to 1) bias (ECT), 2) random disturbance, and 3) deviation of the regression slope from unity (ER) was also examined (Bibby and Toutenburg, 1977; Tedeschi, 2006). The ECT, ED, and ER were then expressed as a percent of MSPE:


$$\begin{array}{l} \begin{array}{l} ECT={\left(\bar{P}-\bar{O}\right)}^{2}\; \end{array} \end{array}$$ (2)

$$\begin{array}{l} \begin{array}{l} ED=\left(1-R^{2}\right)\times \;{\left(S_{o}\right)}^{2}\; \end{array} \end{array}$$ (3)

$$\begin{array}{l} \begin{array}{l} ER={\left(S_{p}-R\times S_{o}\right)}^{2}\; \end{array} \end{array}$$ (4)

where $\bar{P}=Predicted\;mean$, $\bar{O}=Observed\;mean$, R=Pearson correlation coefficient,$S_{p}=Predicted\;standard\;deviation,$$and\;S_{o}=Observed\;standard\;deviation$

To determine the reproducibility index, the concordance correlation coefficient (**CCC**) was calculated (Lin, 1989). The CCC provides a metric in the range −1 to 1, wherein −1 indicates perfect disagreement, 1 indicates perfect agreement, and a 0 value indicates no relationship. Within the CCC equation, a bias correction factor (*C*_*b*_) to measure accuracy is calculated where ν is indicative of scale shift, or change in standard deviation, between the predicted and observed values, and µ is an indication of location shift. An ideal ν value is equal to 1 whereas a value greater than 1 suggests that predicted values have greater variance compared to the observed values. When µ is negative, this indicates over prediction; alternatively, a positive µ value indicates under prediction:


$$\begin{array}{l} \begin{array}{l} CCC=R\times C_{b}\; \end{array} \end{array}$$ (5)

where


$$\begin{array}{l} \begin{array}{l} C_{b}=\frac{2}{\left(v+1)/(v+\mu^{2}\right)}\; \end{array} \end{array}$$ (6)

$$\begin{array}{l} \begin{array}{l} v=\frac{S_{o}}{S_{p}}\; \end{array} \end{array}$$ (7)

$$\begin{array}{l} \begin{array}{l} \mu =\frac{\bar{O}-\bar{P}}{{\left(S_{o}\times S_{p}\right)}^{\frac{1}{2}}}\; \end{array} \end{array}$$ (8)

## 3. Results

Collinearity analyses of the continuous and categorical X variables are summarized in Table 3. Significantly collinear X variables determined using PROC CORR included NFE (% ME) and fat (% ME) and protein (% ME) (*R* = −0.756, *P* < 0.0001 and *R* = −0.511, *P* < 0.0001, respectively). Collinearity was also observed between fat (% ME) and BFM (*R* = 0.373, *P* = 0.0463), DEI and study length (*R* = −0.310, *P* = 0.0303), and between BW and BFM (*R* = 0.753, *P* < 0.0001), and BW and BF% (*R* = 0.548, *P* = 0.0021). A collinear relationship was also observed between BFM and BF% and study length (*R* = 0.951, *P* < 0.0001 and *R* = 0.417, *P* = 0.0244, respectively), and between BF% and study length (*R* = 0.550, *P* = 0.002). The Kruskal–Wallis test identified significant collinear relationships between NFE (% ME) and NFE level, fat level, and protein level (X^2^ (2, *n* = 53) = 40.572, *P* < 0.0001; X^2^ (2, *n* = 53) = 19.401, *P* < 0.0001; and X^2^ (2, *n* = 53) = 10.492, *P* = 0.0053, respectively). Fat (% ME) was observed to be collinear with NFE level and fat level (X^2^ (2, *n* = 53) = 12.754, *P* = 0.0017 and X^2^ (2, *n* = 53) = 38.6812, *P* < 0.0001) and study design (X^2^ (2, *n* = 53) = 5.782, *P* = 0.0162), but not protein level (X^2^ (2, *n* = 53) = 1.406, *P* = 0.5638). Collinearity was observed between protein (% ME) and NFE level (X^2^ (2, *n* = 53) = 18.868, *P* < 0.0001) and protein level (X^2^ (2, *n* = 53) = 34.576, *P* < 0.0001). A significant collinear relationship was observed between BC and all continuous variables except NFE, fat, and protein (% ME) (*P* > 0.05). Collinearity was also observed between feeding method and DEI, BF%, and study length and between study design and BF% and study length (*P* > 0.05). Additionally, fat level and BFM were found to be collinear (X^2^ (2, *n* = 29) = 5.668, *P* = 0.05). Collinearity between categorical variables was observed, where Trade had a significant collinear relationship with feeding method, NFE level and protein level, BC and feeding method were collinear, and collinearity was observed between NFE level and protein- and fat- level (*P* < 0.05).

**Table 3.** Collinearity between continuous and categorical driving variables in the database for the meta-analysis determined using Pearson correlation coefficients (*R*) and Chi-Square tests

Driving variables^1^	NFE, % ME	Fat, % ME	Protein, % ME	DEI, kcal/kg BW	BW, kg	BFM, kg	BF%, %	Study Length
*Pearson correlation coefficients*								
NFE, % ME	1.000	—	—	—	—	—	—	—
Fat, % ME	−0.756^2^	1.000	—	—	—	—	—	—
Protein, % ME	−0.511^2^	−0.113	1.000	—	—	—	—	—
DEI, kcal/kg BW	0.064	−0.010	−0.111	1.000	—	—	—	—
BW, kg	−0.299	0.208	0.139	−0.179	1.000	—	—	—
BFM, kg	−0.275	0.373^2^	0.032	−0.270	0.753^2^	1.000	—	—
BF%, %	−0.189	0.355	−0.045	−0.244	0.548^2^	0.951^2^	1.000	—
Study length^3^	0.314	−0.118	0.069	−0.310^2^	0.153	0.417^2^	0.550^2^	1.000
*Chi-Square*								
Trade^4^	0.396	1.180	1.287	9.494^2^	0.219	0.005	0.002	0.385
BC^5^	0.385	0.029	0.390	8.354^2^	13.760^2^	11.393^2^	6.604^2^	10.355^2^
Study design^6^	1.309	5.782^2^	1.117	1.098	0.148	2.709	4.357^2^	17.550^2^
Feed method^7^	1.134	3.223	2.352	21.160^2^	1.013	5.843	6.061^2^	23.022^2^
NFE level	40.572^1^	12.754^2^	18.868^2^	1.104	1.380	3.191	2.824	0.392
Fat level	19.401^1^	38.681^2^	1.146	1.737	3.679	5.668^2^	4.690	0.959
Protein level	10.492^1^	1.406	34.576^2^	4.506	1.285	0.569	0.288	1.445
	Trade	Study design	BC	Feed method	NFE level	Fat level	Protein level	
*Chi-square*								
Trade	—	—	—	—	—	—	—	
Study design	3.463	—	—	—	—	—	—	
BC	0.888	1.399	—	—	—	—	—	
Feed method	6.099^2^	3.513	5.944^2^	—	—	—	—	
NFE level	6.275^2^	0.270	0.566	3.192	—	—	—	
Fat level	1.107	0.734	0.885	1.489	4.851^2^	—	—	
Protein level	4.430^2^	1.720	0.007	2.995	8.586^2^	3.050	—	

^1^NFE = nitrogen-free extract, ME = metabolizable energy, DEI = daily energy intake, BW = body weight, BFM = body fat mass, BF%=body fat percent, BC = body composition.

^2^ *P* < 0.05.

^3^Study length in weeks.

^4^Trade includes “NFE-Fat”, “NFE-protein”, or “Dilution”.

^5^BC includes “Lean” or “Obese”.

^6^Study Design includes “cross-over” or “parallel”^.^

^7^Feed method includes “ad libitum” or to “maintenance energy requirements”.

Equations for the best-performing models for BFM, fasted insulin (pmol/L), and fasted glucose (mmol/L) are summarized in Table 4 and discussed for each response variable.

**Table 4.** Best performing and best overall fit models for predicting BFM (kg), fasted insulin (pmol/L), and fasted glucose (mmol/L) in the meta-analysis

Equation ID^1^	X variables^2^	Parameter	Estimate	*P*-value^3^
*Body fat mass (kg)*				
EQN-BFM1	NFE, % ME DEI, kcal/kg BW	Intercept:	2.214 (±0.45)	0.0012
		NFE (% ME)* DEI (kcal/kg BW):	−0.00016 (±0.00007)	0.0396
*Fasted insulin (pmol/L)*				
EQN-INS1	NFE, % ME BFM, kg	Intercept:	53.143 (±9.77)	0.0028
		NFE (% ME)*BFM:	−0.290 (±0.15	0.0816
EQN-INS5	NFE, % ME DEI, kcal/kg BW BW, kg	Intercept:	51.639 (±5.78)	<0.0001
		NFE (% ME)*DEI (kcal/kg BW)*BW (kg):	−0.00012 (±0.0003)	0.6882
*Fasted glucose (mmol/L)*				
EQN-GLUC2	NFE, % ME BF%	Intercept:	4.213 (±0.40)	<0.0001
		NFE (% ME):	0.0023 (±0.004)	0.5320
		BF%:	0.017 (±0.01)	0.1237
EQN-GLUC6	NFE Level DEI, kcal/kg BW BW, kg	Intercept:	4.436 (±0.45)	<0.0001
		NFE Level*: High* NFE Level*: Moderate* NFE Level*: Low*	0.339 (±0.12)	0.0292
			0	
			0.242 (±0.12)	
		DEI (kcal/kg BW):	−0.0054 (±0.003)	0.1039
		BW (kg):	0.074 (± 0.08)	0.3695

^1^BFM = body fat mass, EQN = equation.

^2^X = driving variable, NFE = nitrogen-free extract, DEI = daily energy intake (kcal/kg BW), ME = metabolizable energy, BFM = body fat mass, BF%=body fat percent, BW = body weight.

^3^Significance set at *P* < 0.05.

### 3.1. Body fat mass

The univariate models to test the significance of X variables pertaining to dietary macronutrient content in predicting BFM are summarized in Table 5. NFE (continuous), NFE level (categorical), Trade (categorical), and fat (continuous) did not affect the dependent variable (*P* > 0.05). However, fat level (categorical) appeared to affect BFM in cats (*P* = 0.0039). Univariate models for all other X variable univariate analyses are available in Table S1. Of note, study length (continuous, *P* < 0.0001) and study design (categorical, *P* = 0.0139) appeared to affect BFM in cats. Model evaluation parameters suggest that for all univariate models, the majority of error stems from random sources (ED% range, 82.03% to 96.14%). The RMSPE for all models are moderately high (>46.22%), and CCC values are low (<0.59) suggesting poor overall fit of the data.

**Table 5.** Univariate model parameter estimates and fit statistics of each dietary macronutrient driving (X) variable for BFM (kg), fasted insulin (pmol/L), and fasted glucose (mmol/L) prediction via a meta-analysis

X Variable^1^	Parameter	Estimate	*P*-value^2^	*N* ^3^	*R* ^4^	RMSPE, %^5^	ECT, %^6^	ER, %^7^	ED, %^8^	Cb^9^	CCC^10^
*Body Fat Mass, kg*											
NFE, % ME	Intercept	1.227 ± 0.25	0.0009	29	0.67	48.85	2.09	3.95	93.96	0.81	0.54
	NFE	−0.00066 ± 0.002	0.7757								
Fat, % ME	Intercept	0.885 ± 0.38	0.0475	29	0.72	46.42	2.31	6.52	91.17	0.82	0.59
	Fat	0.0099 ± 0.01	0.3157								
Protein, % ME	Intercept	1.194 ± 0.25	0.0014	29	0.66	49.11	2.11	3.63	94.26	0.81	0.53
	Protein	0.00030 ± 0.003	0.9173								
NFE Level	Intercept	1.217 ± 0.43	0.0228	29	0.67	48.87	2.15	3.96	93.89	0.81	0.54
	High	−0.0264 ± 0.42	0.9336								
	Moderate	0									
	Low	0.001 ± 0.42									
Fat Level	Intercept	0.596 ± 0.05	<0.0001	29	0.72	46.23	5.20E^−10^	9.43	90.57	0.80	0.58
	High	0.932 ± 0.35	0.0039								
	Moderate	0									
	Low	0.787 ± 0.27									
Protein Level	Intercept	1.405 ± 0.48	0.0191	29	0.65	49.45	1.91	3.18	94.91	0.81	0.53
	High	−0.207 ± 0.46	0.8777								
	Moderate	0									
	Low	−0.224 ± 0.46									
Trade	Intercept	1.189 ± 0.25	0.0019	29	0.70	48.99	7.23E^−10^	13.11	86.89	0.71	0.50
	NFE-Fat	0.0943 ± 0.61									
	NFE-Protein	0									
	Dilution	-									
*Body Fat Mass, kg:* Subgroup Analysis: Lean cats only											
NFE, % ME	Intercept	1.324 ± 0.19	0.0010	19	0.80	31.35	1.44E^−29^	4.40	95.60	0.92	0.73
	NFE	−0.0154 ± 0.005	0.0053								
Fat, % ME	Intercept	0.154 ± 0.20	0.4739	19	0.82	28.99	3.77E^−10^	2.52	97.48	0.95	0.79
	Fat	0.0216 ± 0.01	0.0011								
Protein, % ME	Intercept	0.902 ± 0.43	0.0871	19	0.57	44.96	2.79E^−29^	14.47	85.53	0.44	0.25
	Protein	−0.0005 ± 0.01	0.9652								
*Fasted Insulin, pmol/L*											
NFE, % ME	Intercept	40.274 ± 4.91	<0.0001	39	0.66	35.98	22.28	7.36	70.36	0.62	0.41
	NFE	0.0401 ± 0.08	0.6299								
Fat, % ME	Intercept	43.051 ± 5.13	<0.0001	39	0.67	35.85	22.50	7.75	69.74	0.62	0.42
	Fat	−0.0474 ± 0.08	0.5716								
Protein, % ME	Intercept	36.840 ± 9.46	0.0025	39	0.69	35.40	23.12	8.64	68.24	0.63	0.43
	Protein	0.132 ± 0.24	0.5910								
NFE Level	Intercept	44.673 ± 5.83	<0.0001	39	0.61	36.62	20.07	4.01	75.92	0.64	0.39
	High	−0.4787 ± 5.69	0.1234								
	Moderate	0									
	Low	−6.367 ± 5.47									
Fat Level	Intercept	35.606 ± 6.25	0.0001	39	0.70	34.01	21.15	8.06	70.79	0.68	0.48
	High	5.063 ± 6.168	0.0608								
	Moderate	0									
	Low	10.831 ± 6.21									
Protein Level	Intercept	31.485 ± 9.12	0.0062	39	0.72	34.18	24.33	9.32	66.36	0.67	0.48
	High	14.768 ± 10.72	0.3771								
	Moderate	0									
	Low	10.847 ± 10.31									
Trade	Intercept	51.288 ± 4.91	<0.0001	39	0.63	36.91	26.04	1.71	72.24	0.69	0.43
	NFE-Fat	−21.315 ± 6.61	0.0119								
	NFE-Protein	−8.680 ± 8.29									
	Dilution	-									
*Fasted Glucose, mmol/L*											
NFE, % ME	Intercept	4.662 ± 0.16	<0.0001	45	0.93	5.34	5.22	18.97	75.80	0.95	0.89
	NFE	−0.0006 ± 0.003	0.8536								
Fat, % ME	Intercept	4.601 ± 0.19	<0.0001	45	0.93	5.27	4.99	19.36	75.65	0.95	0.89
	Fat	0.00133 ± 0.004	0.7407								
Protein, % ME	Intercept	4.758 ± 0.22	<0.0001	45	0.93	5.22	4.50	17.86	77.64	0.96	0.89
	Protein	−0.003 ± 0.005	0.5270								
NFE Level	Intercept	4.557 ± 0.16	<0.0001	45	0.94	4.92	4.58	18.50	76.92	0.96	0.90
	High	0.1461 ± 0.13	0.5131								
	Moderate	0									
	Low	0.102 ± 0.12									
Fat Level	Intercept	4.824 ± 0.15	<0.0001	45	0.92	5.57	7.08	15.93	76.99	0.95	0.88
	High	−0.237 ± 0.11	0.1031								
	Moderate	0									
	Low	−0.245 ± 0.13									
Protein Level	Intercept	4.585 ± 0.18	<0.0001	45	0.92	5.59	5.93	17.27	76.79	0.95	0.87
	High	0.113 ± 0.18	0.7712								
	Moderate	0									
	Low	0.0382 ± 0.16									
Trade	Intercept	4.569 ± 0.21	<0.0001	45	0.93	5.33	5.06	19.03	75.91	0.95	0.89
	NFE-Fat	0.0605 ± 0.29	0.8616								
	NFE-Protein	0.166 ± 0.30									
	Dilution	0									

^1^ **NFE = nitrogen-free extract**, ME = metabolizable energy, DEI = daily energy intake, BW = body weight, Ad Lib = ad libitum, MER = maintenance energy.

Requirements ^2^Significance set at *P* < 0.05 ^3^N = sample size (observations used) ^4^R = Pearson correlation coefficient ^5^Root mean square prediction error expressed as a percentage of the observed mean ^6^Error due to bias expressed as a percentage of MSPE ^7^Error due to regression slope deviation expressed as a percentage of MSPE ^8^Error due to disturbance expressed as a percentage of MSPE ^9^Bias correction factor ^10^Mean concordance correlation coefficient.

The subgroup analysis using only lean cats to build univariate models with NFE (% ME), fat (% ME), and protein (% ME) to predict BFM (kg) are presented in Table 5. Protein (% ME) as the X variable demonstrated poor fit with a low *R* and CCC values of 0.57 and 0.25, respectively. Alternatively, NFE (% ME) and fat (% ME) were observed to have good overall fit with low RMSPE (31.35% and 28.99%, respectively), high *R* values (0.80 and 0.82, respectively), and moderately high CCC values (0.73 and 0.79, respectively). Error from random source was observed to be the major source of error for all 3 univariate models (ED%, > 85.53%). Significance was observed for both NFE (% ME) (*P* = 0.0053) and fat (% ME) (*P* = 0.0011). For every 1% increase of NFE (% ME), BFM predictions were reduced by 0.0154 ± 0.005 kg. A 1% increase in fat (% ME) was determined to increase BFM by 0.0216 ± 0.01 kg.

**Figure 2.**
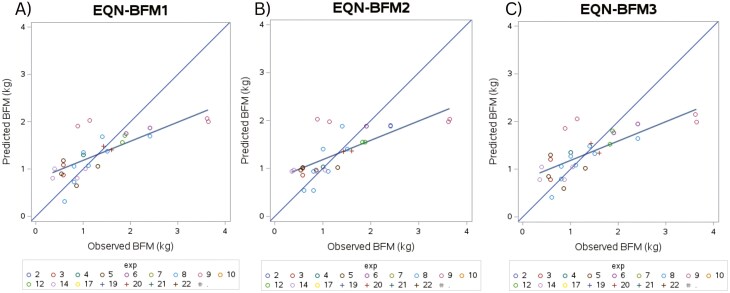
Predicted versus observed plots for the 3 top performing equations to predict BFM (kg). (A) EQN-BFM1, (B) EQN-BFM2, and (C) EQN-BFM3.

A total of 25 multivariate models were built to predict BFM, and the 3 best-performing models are presented in Table 6. Plots of the predicted and observed values are illustrated in Fig. 2 for these models. Error for all 3 models was, in majority, from random source (ED%, 92.58% to 93.47%). Overall, from both the evaluation statistics and use of predicted versus observed plots, these 3 models are considered to have only a moderate fit where RMSPE was low (<45%) but *R* and CCC values are not high; with *R* ranging from 0.73 to 0.74, and CCC values at 0.62 for all 3 models. Of the 3 models, EQN- BFM3 can be considered the best-fitting model via the fit statistics. However, in this model (EQN-BFM3), NFE (% ME) and DEI (kcal/kg BW) were not observed to be significant (*P* = 0.1282 and *P* = 0.2135, respectively). Therefore, EQN- BFM1 was chosen as the best model due to similar fit statistics and observed significance. From this model, as NFE (% ME) or DEI (kcal/kg BW) increases, a decrease in BFM (kg) is observed (−0.00016 ± 0.00007) which was observed to be significant (*P* = 0.0396) (Table 4).

**Table 6.** Evaluation statistics for the best-performing multivariate prediction models for the response variable, BFM (kg) (BFM), fasted insulin (pmol/L) (INS), and fasted glucose (mmol/L) (GLUC) from the meta-analysis where equations 1 to 3 for INS and GLUC include BFM and body fat percent (BF%) as driving (X) variables and equations 4 to 6 for INS and GLUC removed BFM and BF% as potential X variables

Equation^1^	X^2^	*N* ^3^	*R* ^4^	RMSPE, %^5^	ECT, %^6^	ER, %^7^	ED, %^8^	*C*_*b*_ ^9^	CCC^10^
*Body fat mass, kg*									
EQN-BFM1	NFE (% ME)*DEI	29	0.74	44.66	3.48E^−11^	7.42	92.58	0.84	0.62
EQN-BFM2	Trade, Fat (% ME)	29	0.73	44.91	1.38E^−10^	6.53	93.47	0.85	0.62
EQN-BFM3	NFE (% ME), DEI	29	0.74	44.58	3.50E^−11^	7.15	92.85	0.85	0.62
*Fasted insulin, pmol/L*									
EQN-INS1	NFE (% ME)*BFM	19	0.90	20.19	4.18E^−11^	5.19	94.81	0.97	0.87
EQN-INS2	Trade, BF%	19	0.82	25.57	2.61E^−11^	1.87	98.13	0.96	0.79
EQN-INS3	NFE (% ME)*DEI*BFM	19	0.86	23.14	3.18E^−11^	6.53	93.47	0.95	0.82
EQN-INS4	NFE (% ME)*DEI	39	0.91	17.20	4.64E^−11^	3.72	96.28	0.98	0.90
EQN-INS5	NFE (% ME)*DEI*BW	35	0.91	17.18	1.16E^−11^	3.71	96.29	0.98	0.90
EQN-INS6	Trade*DEI	35	0.91	17.27	1.15E^−11^	3.41	96.59	0.98	0.90
Fasted glucose, *mmol/L*									
EQN-GLUC1	Trade, BF%	21	0.94	3.66	3.23E^−09^	2.80	97.20	0.99	0.93
EQN-GLUC2	NFE (% ME), BF%	21	0.95	3.53	3.01E^−27^	2.70	97.30	0.99	0.94
EQN-GLUC3	NFE (% ME)*DEI BF%	21	0.94	3.68	7.99E^−10^	2.83	97.17	0.99	0.93
EQN-GLUC4	NFE Level, DEI	41	0.95	3.98	2.09E^−26^	1.95	98.05	1.00	0.95
EQN-GLUC5	NFE Level*DEI	41	0.95	4.08	1.67E^−10^	2.03	97.97	1.00	0.95
EQN-GLUC6	NFE Level, DEI, BW	41	0.96	3.89	7.34E^−10^	1.87	98.13	1.00	0.95

^1^BFM = body fat mass, EQN = equation.

^2^X = driving variable, NFE = nitrogen-free extract, DEI = daily energy intake (kcal/kg BW), ME = metabolizable energy.

^3^ *N* = sample size (observations used).

^4^ *R* = Pearson correlation coefficient.

^5^Root mean square prediction error expressed as a percentage of the observed mean.

^6^Error due to bias expressed as a percentage of MSPE.

^7^Error due to regression slope deviation expressed as a percentage of MSPE.

^8^Error due to disturbance expressed as a percentage of MSPE.

^9^Bias correction factor.

^10^Mean concordance correlation coefficient.

### 3.2. Fasted insulin

Univariate models for each dietary macronutrient X variable on fasted insulin are outlined in Table 5 and all other univariate models are summarized in Table S1. There was no independent effect of any the continuous X variables on fasted insulin (*P* > 0.05). Trade (categorical, *P* = 0.0119) affected the dependent variable and a trend was observed for fat level (*P* = 0.0608), whereas NFE level and protein level did not affect fasted insulin (*P* > 0.05). Upon model evaluation, univariate models had majority of error from random sources (ED%, 66.36% to 81.05%), low RSMPE (23.92% to 36.91%), and variable CCC values (0.39 to 0.81) indicating poor model fit of data.

Regarding NFE and NFE level as a predictor for fasted insulin in cats, a lack of significance of slope across the univariate models was observed (*P* > 0.05). Additionally, significance was observed for fat level (*P* = 0.0039) where a high-fat level results in a greater increase in fasted insulin concentrations compared to a moderate fat level or a low-fat level; though a low-fat level also increases fasted insulin compared to a moderate fat level. A trend was observed for the univariate model where BFM was the X variable (*P* = 0.0743) such that a 1 kg increase in fat mass increases fasted insulin by 10.95 ± 5.60 pmol/L (S1).

**Figure 3.**
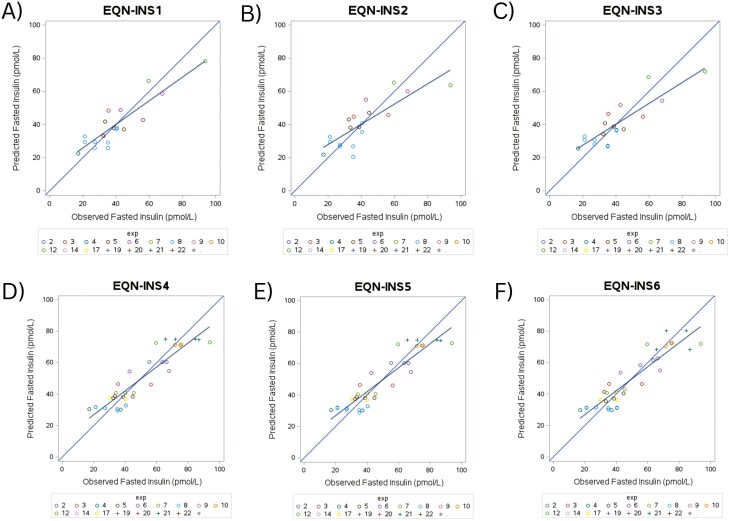
Predicted versus observed plots of the top 3 performing models for fasted insulin (pmol/L) wherein BFM and body fat percent (BF%) were included as driving (X) variables (A–C) and the top 3 performing models for fasted insulin (pmol/L) wherein BFM and BF% were removed as X variables (D–F) in the meta-analysis.

Multivariate models for fasted insulin using the stepwise approach from the univariate models, NFE (% ME), Trade, and NFE level, wherein only X variables that were deemed not to be collinear (Table 3) were added, resulted in 153 total models evaluated. Equations with model fitting errors (fixed or random) were removed resulting in 120 equations. Evaluation of the top 6 performing multivariate equations are reported in Table 6 and the predicted versus observed plots are illustrated in Fig. 3. Models where BFM and BF% were deemed viable X variables (EQN-INS1-3) indicated good overall fit identified by the low RSMPE (20.19% to 25.57%) and high *R* and CCC values (*R*, 0.82 to 0.90 and CCC, 0.82 to 0.87, respectively). Error from random sources appeared to be the majority of the error (ED%, 93.47% to 98.13%). The best 3 performing multivariate models (EQN-INS4-6) wherein BFM and BF% were excluded as X variables due to low study inclusion had overall good fit according to evaluation fit statistics. All 3 equations had low RSMPE between 17.18% and 17.27%, and all 3 had a high *R* value of 0.91 and CCC value of 0.90. Error for all 3 equations stemmed from mostly random sources (ED%, 96.28% to 96.29%). EQN-INS1 and EQN-INS5 were considered the best performing, and best overall fit, models (Table 4). From EQN-INS1, a trend was observed for the interaction of NFE (% ME) and BFM as the fixed effects in the prediction model for fasted insulin (pmol/L) (*P* = 0.0816). Specifically, an increase in NFE (% ME) and/or BFM suggests a decrease in fasted insulin concentration (pmol/L) in cats. Alternatively, no significance was observed for the fixed effects, NFE (% ME), DEI, and BW, in EQN-INS5 (Table 4).

### 3.3. Fasted glucose

Each dietary macronutrient X variable univariate models assessed for the dependent variable, fasted glucose, are presented in Table 5. No significance (*P* > 0.05) was observed for prediction of fasted glucose with any individual X variable tested. According to evaluation statistics, all univariate models have a good overall fit with low RSMPE (<6%), high *R* (>0.90), and high CCC (>0.86) with majority of error coming from random sources (ED%, >75%).

**Figure 4.**
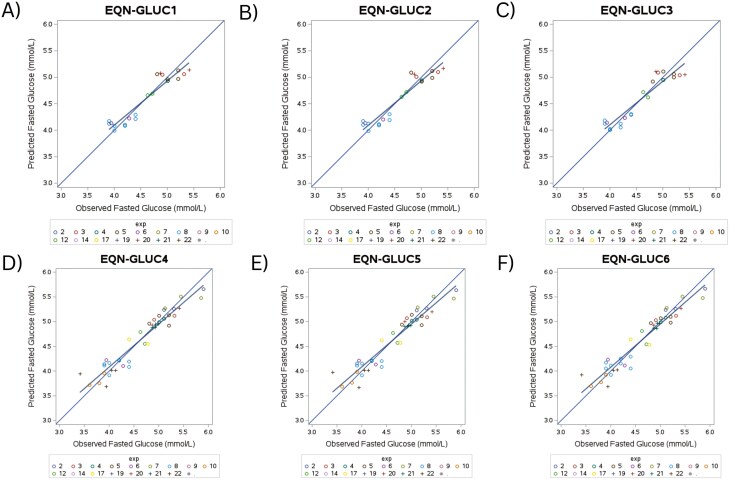
Predicted versus observed plots of the top 3 performing models for fasted glucose (mmol/L) wherein BFM and body fat percent (BF%) were included as driving (X) variables (A–C) and the top 3 performing models for fasted glucose (mmol/L) wherein BFM and BF% were removed as X variables (D–F) in the meta-analysis.

A total of 153 multivariate models were tested for fasted glucose when using a stepwise approach building from the following univariate models: NFE (% ME), Trade, or NFE level. Seven models were excluded due to model fitting errors resulting in 146 models. When BFM and BF% were included as X variables, 3 best-performing equations (EQN-GLUC1-3, Table 6) were observed to have good overall fit. The predicted versus observed plots for EQN-GLUC1 through 6 are illustrated in Figure 4. Upon evaluation of fit statistics, EQN-GLUC2 was observed to be the best-performing model with best overall fit wherein there was a low RMSPE (3.53%), and high *R* and CCC values of 0.95 and 0.94, respectively. Similarly, the 3 best-performing equations (EQN-GLUC4-6, Table 6) indicated good overall fit. When BFM and BF% were removed as X variables, EQN-GLUC6 appeared to be the best-performing model such that the RSMPE was equal to 3.89% and the highest *R* and CCC values between EQN-GLUC4-6 of 0.96 and 0.95, respectively. For all models (EQN-GLUC1-6), random source was observed to be the largest contributor to error (ED%, 97.17% to 98.13%).

EQN-GLUC2 and EQN-GLUC6 were considered the best overall models for prediction of glucose within the meta-analysis (Table 4). For EQN-GLUC2, an increase of 1% NFE (% ME) increases fasted glucose by 0.290 (±0.15) and an increase of 1% BF results in 0.017 (±0.01) increase in fasted glucose (mmol/L); though there was no significance observed for the fixed effects, NFE and BF% (*P* > 0.05). Fasted glucose concentrations were predicted to increase significantly with changes in NFE level, such that a high or low NFE level resulted in increased fasted glucose, but not when NFE level was considered moderate (*P* = 0.0292), with DEI and BW as covariates within the fixed effect model.

## 4. Discussion

This meta-analysis aimed to investigate the effects of dietary carbohydrates, as NFE, on BFM as well as fasted insulin and glucose concentrations in cats. To the authors’ knowledge, this is the first meta-analysis to examine the role of dietary carbohydrates on these physiological outcomes in domestic cats. Regarding BFM, several studies have sought to understand how differing macronutrient contents can affect both BW and BFM gains (Nguyen et al., 2004; Backus et al., 2007; Martin et al., 2010; Wei et al., 2011; Coradini et al., 2014; Gooding et al., 2015). Macronutrient levels are considered as high (NFE: > 30% ME; Fat: > 35% ME; Protein: > 42% ME), moderate (NFE: 24% ME—30% ME; Fat: 28% ME—35% ME; Protein: 34% ME—42% ME), or low (NFE: < 24% ME; Fat: < 28% ME; Protein: < 34% ME) based on relativity to test diets used throughout the available studies. When fed ad libitum, high-fat diets consistently resulted in greater energy intakes compared to high-carbohydrate diets, driving an increase in BW in cats (Nguyen et al., 2004; Backus et al., 2007; Martin et al., 2010; Farrow et al., 2013). The increased BW is largely influenced by an increase in BFM (Nguyen et al., 2004). The effects of high-protein diets compared to high-carbohydrate and high-fat diets on energy intake, BW, and BFM have been conflicting, with one study observing an increase in energy intake with high-protein versus high-fat diets (Farrow et al., 2013), and another observing no difference between high-protein and high-fat diets (Martin et al., 2010). Unfortunately, Farrow et al., did not measure BC; however, Martin et al., observed that cats consuming the high-fat diet had a trend towards increasing BW which was not observed with the high-protein diet (Martin et al., 2010; Farrow et al., 2013).

From the meta-analysis, dietary protein did not appear to influence BFM whereas NFE and fat had opposing effects. From the present study, in lean cats, increasing the NFE concentrations in the food appears to lower the BFM prediction whereas an increase in dietary fat concentrations is likely to increase the BFM. When both lean and obese cats were included in the analysis, these observations were no longer observed; though diets considered high in fat (>35% ME) were observed to influence BFM of cats (EQN-BFM2). These differences in findings could be attributed to the limited dataset. For example, a restricted feeding method was only utilized in obese cats (Laflamme and Hannah, 2005; des Courtis et al., 2015). This could also explain the significance of feed method on BFM in the univariate model wherein ad libitum and MER feeding methods appeared to reduce BFM compared to restricted feeding as the cats on a restricted feeding method were consistently obese, having a greater BFM compared to cats fed to MER or ad libitum (Laflamme and Hannah, 2005; des Courtis et al., 2015). Similar biases could also explain the significance of study length and design on the response variable, BFM. Studies with longer duration, specifically under ad libitum feeding methods, will likely result in greater propensity for both BW and BFM gain compared to shorter durations. Additionally, cross-over study designs carry over any BFM gains from the first period into the next, and so on. Regarding NFE (% ME), the findings from the present study appear to be consistent with previous research in cats, wherein increasing the dietary carbohydrate content of the diet does not result in greater propensity for BFM (Nguyen et al., 2004; Backus et al., 2007; Martin et al., 2010; Gooding et al., 2015). Indeed, the best-fitting model for BFM indicated an interaction between NFE (% ME) and DEI (kcal/kg BW) (EQN-BFM1). This suggests that the proportion of energy from NFE is important. When considering the univariate models, specifically, fat level (categorical), wherein the greater the fat level the greater the increase in BFM, it could be that by increasing NFE (% ME) and thus, its proportion of the DEI, the fat (% ME) is reduced. However, the meta-analysis is limited to a focus on NFE. Therefore, definitive conclusions on the role of dietary fat content cannot be made. Further, this model suggests that an increase in DEI would lead to a reduction in BFM which is unexpected. However, BFM is one component of BW and although an increase in DEI may result in a decrease in BFM this does not necessarily equate to a decrease in overall BW. Important to note is that the reduction in BFM according to the model was estimated to be −0.00016 ± 0.00007 kg for every kcal/kg BW which would not be considered clinically relevant. Additionally, the best-performing models had only a moderate overall fit as determined via the low RMSPE and only moderate CCC and *R* values and confirmed from predicted vs. residual plots. The moderate model fit could be due to the limited sample size, as well as the inability to separate based on sex, neuter status, and age; all of which have previously been associated with BFM risks (Lund et al., 2005; Colliard et al., 2009; Rowe et al., 2017). Further, since the models included studies of various feeding methods, such as fed to MER, restricted feeding, and ad libitum feeding, with the majority of studies utilizing the MER feeding method. Feeding to MER is unlikely to result in BFM gain, though this has been observed previously (Gooding et al., 2015). Regardless, due to the moderate fit of the predictive equations, these should be interpretated with caution, and revisited as the available literature grows.

Similarly, NFE was not observed to affect fasted insulin concentrations in cats in this meta-analysis. It has previously been postulated that dietary protein, specifically amino acids, could have a greater role in triggering insulin secretion in cats (Curry et al., 1982; Verbrugghe and Hesta, 2017). Cats, as obligate carnivores, rely more heavily on amino acids as a glucose source via gluconeogenesis, though they do have the ability to adjust gluconeogenesis rates in response to carbohydrate intake when protein requirements are met (Hoenig et al., 2007a; Green et al., 2008; Case et al., 2010). Previous hypotheses for the development of IR in cats suggested that the long-term consumption of high-carbohydrate diets leads to exhaustion of β cells due to hyperglycemia and ultimately, loss of β cell insulin secretion (Rand et al., 2004), which was observed in cats when hyperglycemia (30 mmol/L) was induced over a 10-d period (Zini et al., 2009). However, the level of glucose administration was far above normal physiological conditions given the level of dietary carbohydrates in cat foods.

The present study is in agreement with the hypothesis that high NFE content in cat foods does not affect fasted insulin concentrations. Overall, prediction models developed for fasted insulin as the Y variable fit the data well based on model fit statistics and predictive vs. residual plots. Interestingly, from the univariate model using Trade as the X variable, when diets differed between NFE and fat concentrations, this appeared to influence fasted insulin outcomes. When assessed on its own, it is not clear whether a replacement of fat by NFE, or a replacement of NFE by fat is the driver for this observation. Considering the univariate models using NFE level and fat level as X variables, it appears that there is a pattern towards reducing fasted insulin with diets considered high (>30% ME) and low (<24% ME) in NFE, though not significantly, whereas high (>35% ME) and low (>28% ME) fat diets trend towards increasing the fasted insulin concentrations. Multivariate models using NFE (% ME) are in agreement with the NFE level findings, wherein increasing NFE has a pattern towards reducing fasted insulin. Additionally, these findings from the univariate models may also explain the observation of Trade as an X variable in 2 of the best-fitting models for predicting BFM (EQN-INS2 and EQN-INS6).

Although EQN-INS5 was not significant, a 3-way interaction between NFE (% ME), DEI (kcal/kg BW), and BW (kg) was one of the best-fitting models for fasted insulin. It is important to note that all 3 variables are connected; NFE as a % ME will be a proportion of the energy intake (kcal), and DEI as kcal/kg BW removes the total BW as a factor—standardizing the energy intake of cats within the model, regardless of size—though increasing BW (kg) will increase the total amount of energy intake (kcal/d). When fed to MER, cats of light BW have a greater DEI (kcal/kg BW) than heavy cats and this may be due to BC differences between groups (Bermingham et al., 2010). Altogether, it indicates a complex relationship where the overall energy intake and BW, and potentially, lean body mass, may modulate the effect of NFE on insulin concentrations. However, studies also included different feeding methods such as MER, restriction, and ad libitum feeding. A true interpretation of this interaction may not be possible due to the various limitations of the meta-analysis; however, it supports that diet composition, BW, and energy intakes alter metabolic responses which needs to be further investigated in cats.

Regarding EQN-INS1, a trend of the interaction between NFE (%) and BFM (kg) as the X variables towards decreasing fasted insulin was observed. Additionally, BFM as the X variable for the univariate model was observed to have a trend towards increasing the fasted insulin concentrations in cats, and BFM, according to univariate models in the meta-analysis, was largely influenced by dietary fat concentrations, and has been confirmed in previous studies in cats (Nguyen et al., 2004; Backus et al., 2007; Martin et al., 2010). A limitation to this meta-analysis is that the primary focus is on NFE and cannot discuss the true role of dietary fats on insulin concentrations in cats. Indeed, these findings suggest that there could be a direct and indirect (via BFM) effect of dietary fat on insulin concentrations on cats; however, a meta-analysis specific to dietary fat effects on these outcomes is needed.

Interestingly, none of the X variables within the meta-analysis were observed to have significance on fasted glucose concentrations. Multivariate models also indicated no significance though overall fit of the predictive equations was considered to be of good fit based on the low RMSPE and high CCC and *R* values as well as the predictive vs. residual plots. That said, EQN-GLUC6 suggests that low and high NFE levels and greater BW increase fasted glucose concentrations, whereas DEI (kcal/kg BW) has a reducing effect. This may be due to the interplay of dietary protein and fat on blood glucose concentrations in cats and could be why the Trade X variable was observed in the 6 best-fitting models for fasted glucose (EQN-GLUC1). Compared to omnivorous species such as humans, or even dogs, cats have alterations and limitations in their metabolic propensity to digest, absorb, and metabolize glucose (Verbrugghe et al., 2012). For production of glucose, cats appear to preferentially utilize the energy source of protein to maintain their blood glucose levels (MacDonald et al., 1984; Zoran, 2002); however, Hoenig et al., demonstrated that cats can utilize glycolysis in a fasted state for blood glucose by using trace glucose with a euglycemic hyperinsulinemic clamp and indirect calorimetry (Hoenig et al., 2006, 2007b). Regardless, the response to increases in glucose appears to be fast-acting, though the clearance of glucose from the blood is slower compared to other species (Verbrugghe et al., 2009). That said, glucose clearance and return to baseline was observed at 90 min post intravenous administration (1 g glucose/kg BW) (Verbrugghe et al., 2009). Thus, it may not be surprising that there was no influence of X variables on fasted glucose concentrations here.

Indeed, assessing only the fasted concentrations of insulin and glucose concentrations poses a limitation to the interpretation of results from this meta-analysis. As the first meta-analysis to the authors’ knowledge investigating dietary carbohydrates on insulin sensitivity, the findings provide a preliminary assessment of the available literature. Due to a dearth of data available and the varying methodologies (intravenous glucose tolerance test, euglycemic clamp, postprandial meal-tests) used to determine circulating insulin and glucose response, this meta-analysis was limited to only fasted insulin and glucose for consistency. Fasted levels can still identify alterations in both glucose tolerance and insulin sensitivity. IR can be characterized by hyperglycemia and hyperinsulinemia, indicating an inability of insulin to bind to insulin receptors (Feldhahn et al., 1999; Rand and Marshall, 2005; Nelson and Reusch, 2014) which can be observed within the fasted state. However, alterations in the fasted insulin and glucose concentrations in cats is more commonly observed when β cell dysfunction has already occurred, and is therefore, a long-term effect (Ferrannini, 1997; Appleton et al., 2001). It should be noted that the mean study length for the studies included in this meta-analysis was 9 ± 6.7 wk, which may seem quite long, but may still not have been long enough to exert this level of effect on insulin and glucose. However, postprandial data could be beneficial in furthering the understanding of the acute effects of macronutrients and insulin sensitivity and glucose tolerances, though currently, studies are generally conflicting. High-carbohydrate diets (NFE, 47% ME; protein, 25% ME; fat 26% ME) were shown to increase postprandial glucose curves with a tendency to increase the postprandial insulin curve in lean cats compared to high-protein (protein, 46% ME; NFE, 27% ME) and high-fat diets (fat, 47% ME; NFE, 26% ME)) (Farrow et al., 2013). Alternatively, a low carbohydrate, high-protein (NFE, 21% ME; protein, 48% ME) versus high-carbohydrate, low protein (NFE, 47% ME; protein, 28% ME) diet resulted in no changes in glucose tolerance or insulin sensitivity in lean cats (Leray et al., 2006). Similar findings have been repeated with high-protein versus high-carbohydrate diets (Hoenig et al., 2007a). Ultimately, a more comprehensive investigation could aid in clarifying the role of dietary carbohydrates on insulin and glucose responses in cats.

Additional limitations of the meta-analysis include the use of NFE as a measure of dietary carbohydrates. Although this is consistent with the current literature, NFE can include simple sugars, starches, and soluble fibers. Although extruded dry foods are thought to be composed of mostly starches to aid in structure and texture of the kibble (Case et al., 2010; Moser et al., 2011), the true carbohydrate distribution has not been investigated. Additionally, the role of fiber on BW and BFM gain, obesity, IR, and diabetes mellitus in cats is not well understood. However, the source of carbohydrates and both insoluble and soluble fibers could be important in the glucose and insulin responses in cats (Verbrugghe et al., 2012). Many of the best fitting models included an interaction between NFE (% ME) and DEI, highlighting the relationship between dietary NFE content and its contribution to total energy intake. This approach was chosen to facilitate direct comparisons between high and low carbohydrate diets across various studies. Using % ME as a measure of NFE also allowed for the incorporation of the Trade variable, which considers the effects of NFE replacing fat, protein, or a combination of both. This is crucial because increasing NFE content inevitably leads to a decrease in fat or protein. However, another valuable method to consider in future investigations is the total NFE intake (g/kg BW). Other considerations can be made to the various methodologies such as feeding regimen (i.e., restriction, ad libitum, MER), study lengths, and importantly, insulin and glucose concentrations were measured in plasma (Thiess et al., 2004; Backus et al., 2007; Martin et al., 2010; Coradini et al., 2011; Tan et al., 2011; Deng et al., 2013; Farrow et al., 2013; Gooding et al., 2014) or serum (Verbrugghe et al., 2010; Wei et al., 2011; Gooding et al., 2015), or whole blood for glucose (Martin et al., 2010; Deng et al., 2013). Also, the effect of sex and neuter status could not be considered in models within the meta-analysis as many studies included grouped means without separating for these variables. Males have previously been proposed to have a greater propensity for obesity (Lund et al., 2005; Colliard et al., 2009; Courcier et al., 2012; Rowe et al., 2015) as well as IR and diabetes mellitus (Panciera et al., 1990; McCann et al., 2007; Prahl et al., 2007; Kienzle and Moik, 2011; Öhlund et al., 2017). Moreover, gonadectomized cats are also more at risk for the development of obesity, IR, and diabetes mellitus (Panciera et al., 1990; Scarlett et al., 1994; Lund et al., 2005; McCann et al., 2007; Prahl et al., 2007; Colliard et al., 2009). Last, due to the link between obesity and IR, the inclusion of both obese and lean cats could be considered a potential limitation; though subgroup analyses resulted in small sample sizes that could jeopardize the validity of the meta-analysis approach. As increasing research is conducted, this should be re-investigated.

The findings from this meta-analysis agree with the hypotheses that increasing NFE (% ME) does not increase BFM, fasted insulin, or fasted glucose in cats. Future research should aim to further explore the postprandial responses of insulin and glucose to different dietary carbohydrate concentrations. Additionally, this meta-analysis suggests that investigations into dietary fat as a driver for BFM and thus IR should be further explored. Dietary protein did not appear to be a catalyst for any response variables tested, though a more comprehensive analysis specifically into proteins is justified due to the role of protein, specifically amino acids, as a glucose source for cats and a potent stimulator of insulin secretion.

## Supplementary Material

skaf071_suppl_Supplementary_Table_1

## Abbreviations

AAFCO: Association of American Feed Control Officials
AICc: corrected Akaike information criteria
BC: body composition
BFM: body fat mass
BF%: body fat percent
BW: body weight
CCC: concordance correlation coefficient
DEI: daily energy intake
EQN: equation
IR: insulin resistance
MPSE: mean square prediction error
ME: metabolizable energy
NFE: nitrogen-free extract
RMPSE: root mean square prediction error
X: driving variable
