# Supplementary material for: A meta-analysis: dietary carbohydrates do not increase body fat or fasted insulin and glucose in cats
Source: J Anim Sci. 2025 Mar 7;103:skaf071. doi: 10.1093/jas/skaf071 (PMC12010702; doi:10.1093/jas/skaf071)
Supplement: skaf071_suppl_Supplementary_Table_1 [file skaf071_suppl_supplementary_table_1.docx]

| **Supplementary Table 1.** Univariate model parameter estimates and fit statistics of each driving (X) variable for body fat mass (BFM, kg), fasted insulin (pmol/L), and fasted glucose (mmol/L) predictions via a meta-analysis. | | | | | | | | | | | | |
| --- | --- | --- | --- | --- | --- | --- | --- | --- | --- | --- | --- | --- |
| **X Variable^1^** | **Parameter** | **Estimate** | **P-Value^2^** | **N^3^** | **R^4^** | **RMSPE, %^5^** | **ECT, %^6^** | **ER, %^7^** | **ED, %^8^** | | **Cb^9^** | **CCC^10^** |
| ***Body Fat Mass, kg*** | | | | | | | | | | | | |
| DEI, kcal/kg BW | Intercept | 1.601 ± 0.50 | 0.0128 | 29 | 0.71 | 46.84 | 2.93 | 4.38 | 92.69 | | 0.84 | 0.59 |
|  | DEI | -0.0071 ± 0.008 | 0.3933 |  |  |  |  |  |  |  |  |  |
| Study Length | Intercept | 0.348 ± 0.06 | 0.0006 | 29 | 0.64 | 50.16 | 2.48E^-10^ | 4.03 | 95.97 | | 0.78 | 0.49 |
|  | Study Length | 0.057 ± 0.01 | <0.0001 |  |  |  |  |  |  |  |  |  |
| Feed Method | Intercept | 1.524 ± 0.30 | 0.0014 | 29 | 0.18 | 69.14 | 11.86 | 6.11 | 82.03 | | 0.67 | 0.12 |
|  | Ad Lib | -0.265 ± 0.40 | 0.0038 |  |  |  |  |  |  |  |  |  |
|  | MER | -0.930 ± 0.30 |  |  |  |  |  |  |  |  |  |  |
|  | Restrict | 0 |  |  |  |  |  |  |  |  |  |  |
| Study Design | Intercept | 0.978 ± 0.17 | 0.0006 | 29 | 0.60 | 52.16 | 3.85 | 0.01 | 96.14 | | 0.86 | 0.51 |
|  | Cross-Over | 1.306 ± 0.49 | 0.0139 |  |  |  |  |  |  |  |  |  |
|  | Parallel | 0 |  |  |  |  |  |  |  |  |  |  |
| ***Fasted Insulin,* *pmol/L*** | | | | | | | | | | | | |
| DEI, kcal/kg BW | Intercept | 36.002 ± 7.07 | 0.0005 | 35 | 0.70 | 36.18 | 25.11 | 8.35 | 66.54 | | 0.64 | 0.45 |
|  | DEI | -0.0041 ± 0.004 | 0.3188 |  |  |  |  |  |  |  |  |  |
| BW, kg | Intercept | 35.075 ± 10.75 | 0.0076 | 39 | 0.67 | 35.83 | 22.97 | 7.82 | 69.21 | | 0.62 | 0.42 |
|  | BW | 1.373 ± 2.15 | 0.5290 |  |  |  |  |  |  |  |  |  |
| BFM, kg | Intercept | 30.120 ± 9.26 | 0.0226 | 19 | 0.85 | 25.75 | 3.02E^-11^ | 5.23 | 94.77 | | 0.95 | 0.81 |
|  | BF% | 0.095 ± 0.31 | 0.7632 |  |  |  |  |  |  |  |  |  |
| BF%, % | Intercept | 26.333 ± 8.24 | 0.0241 | 19 | 0.85 | 23.93 | 1.11E^-10^ | 6.01 | 63.99 | | 0.95 | 0.81 |
|  | BF% | 0.095 ± 0.31 | 0.7632 |  |  |  |  |  |  |  |  |  |
| Study Length | Intercept | 52.023 ± 6.41 | <0.0001 | 39 | 0.63 | 34.89 | 15.55 | 3.39 | 81.05 | | 0.70 | 0.44 |
|  | Study Length | -1.316 ± 0.62 | 0.0444 |  |  |  |  |  |  | |  |  |
| Feed Method | Intercept | 64.340 ± 20.93 | 0.0118 | 39 | 0.68 | 34.20 | 20.42 | 4.80 | 74.78 | | 0.71 | 0.48 |
|  | Ad Lib | -28.459 ± 21.99 | 0.3962 |  |  |  |  |  |  | |  |  |
|  | MER | -22.248 ± 21.43 |  |  |  |  |  |  |  | |  |  |
|  | Restrict | 0 |  |  |  |  |  |  |  | |  |  |
| BC | Intercept | 59.011 ± 14.99 | 0.0034 | 39 | 0.65 | 34.75 | 19.56 | 2.63 | 77.81 | | 0.73 | 0.47 |
|  | Lean | -19.186 ± 15.72 | 0.4810 |  |  |  |  |  |  | |  |  |
|  | Obese | 0 |  |  |  |  |  |  |  | |  |  |
|  | Mixed | -18.850 ± 18.39 |  |  |  |  |  |  |  | |  |  |
| Study Design | Intercept | 41.053 ± 5.20 | <0.0001 | 39 | 0.68 | 35.47 | 22.26 | 8.58 | 69.16 | | 0.63 | 0.43 |
|  | Cross-Over | 1.158 ± 9.23 | 0.9011 |  |  |  |  |  |  | |  |  |
|  | Parallel | 0 |  |  |  |  |  |  |  | |  |  |
| ***Fasted Glucose,* *mmol/L*** | | | | | | | | | | | | |
| DEI, kcal/kg BW | Intercept | 4.8541± 0.25 | <0.0001 | 41 | 0.93 | 5.38 | 4.91 | 18.62 | 76.46 | | 0.96 | 0.89 |
|  | DEI | -0.0041 ± 0.004 | 0.3188 |  |  |  |  |  |  | |  |  |
| BW, kg | Intercept | 4.2974 ± 0.46 | <0.0001 | 45 | 0.93 | 5.25 | 5.26 | 18.28 | 76.46 | | 0.96 | 0.89 |
|  | BW | 0.0762 ± 0.10 | 0.4326 |  |  |  |  |  |  | |  |  |
| BFM, kg | Intercept | 4.5522 ± 0.23 | <0.0001 | 21 | 0.94 | 3.71 | 3.14E^-09^ | 2.97 | 97.03 | | 0.99 | 0.93 |
|  | BFM | 0.1404 ± 0.11 | 0.2406 |  |  |  |  |  |  | |  |  |
| BF%, % | Intercept | 4.364 ± 0.26 | <0.0001 | 21 | 0.94 | 3.62 | 2.87E^-27^ | 2.81 | 97.19 | | 0.99 | 0.94 |
|  | BF% | 0.0116 ± 0.007 | 0.1397 |  |  |  |  |  |  | |  |  |
| Study Length | Intercept | 4.6855 ± 0.19 | <0.0001 | 45 | 0.93 | 5.38 | 5.37 | 18.10 | 76.53 | | 0.95 | 0.88 |
|  | Study Length | -0.0052 ± 0.018 | 0.7781 |  |  |  |  |  |  | |  |  |
| Feed Method | Intercept | 4.761 ± 0.37 | <0.0001 | 45 | 0.93 | 5.29 | 5.02 | 18.58 | 76.40 | | 0.95 | 0.89 |
|  | Ad Lib | -0.205 ± 0.42 | 0.8218 |  |  |  |  |  |  | |  |  |
|  | MER | -0.112 ± 0.40 |  |  |  |  |  |  |  | |  |  |
|  | Restrict | 0 |  |  |  |  |  |  |  | |  |  |
| BC | Intercept | 4.511 ± 0.28 | <0.0001 | 45 | 0.93 | 5.45 | 5.97 | 19.02 | 75.01 | | 0.95 | 0.88 |
|  | Lean | 0.104 ± 0.32 | 0.3629 |  |  |  |  |  |  | |  |  |
|  | Obese | 0 |  |  |  |  |  |  |  | |  |  |
|  | Mixed | 0.711 ± 0.51 |  |  |  |  |  |  |  | |  |  |
| Study Design | Intercept | 4.750 ± 0.17 | <0.0001 | 45 | 0.93 | 5.34 | 4.67 | 19.53 | 75.80 | | 0.95 | 0.89 |
|  | Cross-Over | -0.234 | 0.3613 |  |  |  |  |  |  | |  |  |
|  | Parallel | 0 |  |  |  |  |  |  |  | |  |  |
| ^1^CHO=carbohydrate, ME=metabolizable energy, DEI=daily energy intake, BW=body weight, Ad Lib=ad libitum, MER=maintenance energy  Requirements ^2^Significance set at P<0.05 ^3^N=sample size (observations used) ^4^R=Pearson correlation coefficient ^5^Root mean square prediction error expressed as a percentage of the observed mean ^6^Error due to bias expressed as a percentage of MSPE ^7^Error due to regression slope deviation expressed as a percentage of MSPE ^8^Error due to disturbance expressed as a percentage of MSPE ^9^Bias correction factor ^10^Mean concordance correlation coefficient | | | | | | | | | |  |  |  |
